# Bilateral Pulmonary Embolism in a Newly Diagnosed Case of Pulmonary Tuberculosis

**DOI:** 10.7759/cureus.12824

**Published:** 2021-01-20

**Authors:** Urooj Zahra, Ali Akhtar, Noor Ul Falah, Shahnawaz Hashmi

**Affiliations:** 1 Internal Medicine, Fatima Jinnah Medical University, Lahore, PAK; 2 Internal Medicine, Army Medical College, Rawalpindi, PAK; 3 Internal Medicine, King Edward Medical University, Lahore, PAK; 4 Internal Medicine, King Edward Medical University, Mayo Hospital, Lahore, PAK

**Keywords:** pulmonary tuberculosis, pulmonary embolism, venous thromboembolism (vte)

## Abstract

Pulmonary embolism in the cases of tuberculosis (TB) infection has incited physicians and researchers to derive a reasonable association in the past few years. However, despite the efforts, pulmonary embolism is often misdiagnosed in the context of active TB. Pulmonary embolism in TB is still considered a rare phenomenon, even though it seems to be a probable risk factor based on the emerging literature. We report a case of a young man who presented with recurrent respiratory symptoms. He had no risk factors except a former history of smoking. We believe that the symptoms he had on the initial visit were misinterpreted in the background of TB infection. We followed the case for two months after he was discharged on anticoagulants and anti-TB medication. In this case report, we would like to emphasize the need to consider this occult phenomenon in order to rule out TB in cases of pulmonary embolism and vice versa. We hope that the management of TB in the future might take into account the chances of concomitant pathology of pulmonary embolism in patients with active TB.

## Introduction

The majority of the developing nations worldwide are affected by tuberculosis (TB) infection every year. It is still considered among the top 10 causes of death worldwide [1]. The infection is caused mainly by Mycobacterium tuberculosis, which is spread through respiratory droplets from one person to another through coughing [2]. Pakistan falls in the list of 30 countries with a high burden of TB infection with a total incidence of TB reported as 265 patients per 100,000 population [2]. Considering the need for venous thromboembolism (VTE) prophylaxis in case of TB infection seems to be imperative [3]. Pulmonary embolism carries a high mortality risk, particularly in patients with a background of chronic infection, heart diseases, or stroke. Emerging data have reported a few cases addressing the need to evaluate pulmonary embolism or provide VTE prophylaxis in patients with TB infection [4].

## Case presentation

A 37-year-old male recently diagnosed with lung TB infection presented with worsening dyspnea and a vague history of dull, right-sided leg pain. The leg pain preceded respiratory distress by approximately one week. It was gradual in onset, 6/10 on the pain scale, constant, dull in character, non-radiating, and did not follow any day or night pattern. He denied any limb weakness, paresthesia, swelling, fever, productive cough, or chest pain. The patient had no co-morbidities in the background apart from a newly acquired pulmonary TB infection. He was a former smoker; quit smoking about 20 years ago. Cardiopulmonary and limb examination were unremarkable.

About a fortnight earlier, he presented with similar symptoms. At that time, high-resolution computerized tomography (HRCT) chest demonstrated a right-sided large pleural effusion with left upper lobe infiltrate and a small left basal patchy infiltrate. The patient was negative for Coronavirus on a qualitative polymerase chain reaction (PCR). Pleural fluid was exudative with lymphocytic background and raised LDH, suggestive of empyema (protein 4.9 g/dl; albumin 2.3 g/dl; WBCs 2039 /cmm; lymphocytes 91%; neutrophils 9%; glucose 80 mg/dl; LDH 851 U/L; RBCs 3000 /cmm). Video-assisted thoracoscopic surgery (VATS) was performed with a right-sided pleural biopsy which showed necrotizing chronic granulomatous inflammation consistent with TB infection. Ziehl-Neelsen stain was positive for acid-fast bacilli. Hence, anti-tubercular therapy was commenced. The patient had no risk factors for immune compromise.

**Figure 1:**
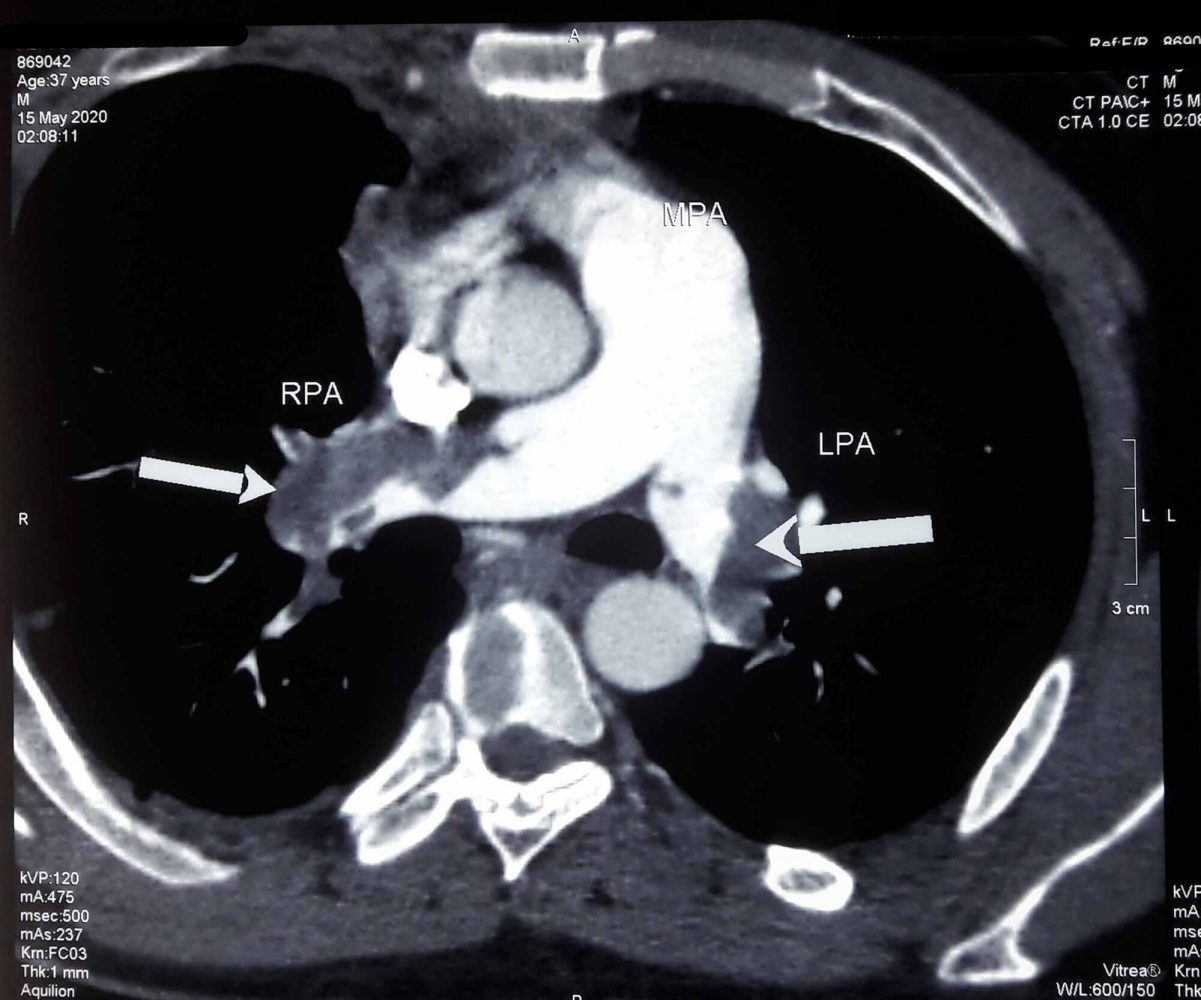
Computed tomographic pulmonary angiography. RPA (white arrow) - occlusive pulmonary thromboembolus in right pulmonary artery; LPA (white arrow) - occlusive pulmonary thromboembolus in left pulmonary artery.

**Figure 2:**
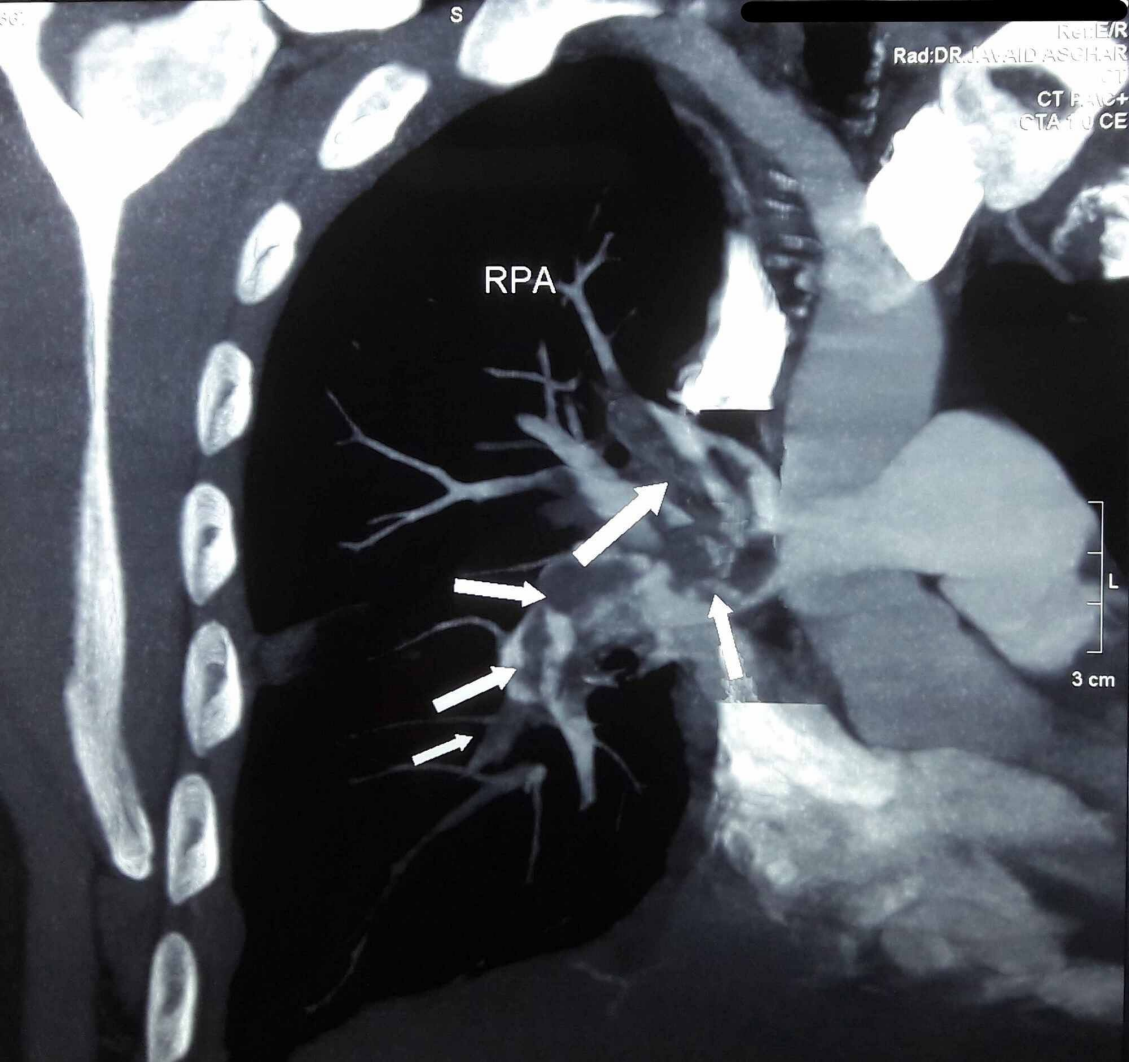
Computed tomographic pulmonary angiography. RPA - right pulmonary artery - occlusive pulmonary thromboembolus involving right pulmonary artery and subsequent branches.

**Figure 3:**
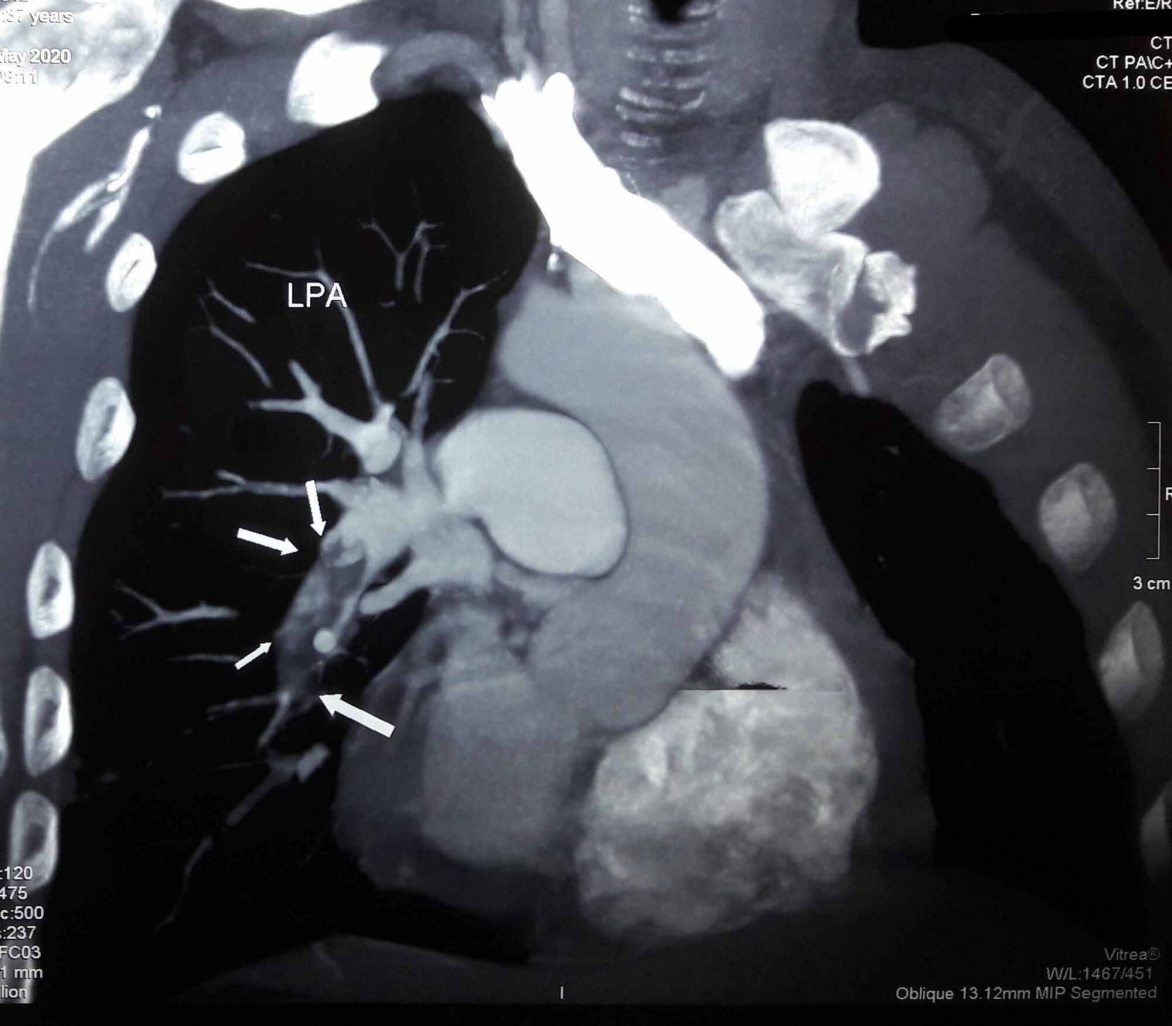
Computed tomographic pulmonary angiography. LPA - left pulmonary artery - occlusive pulmonary thromboembolus involving left pulmonary artery.

**Figure 4:**
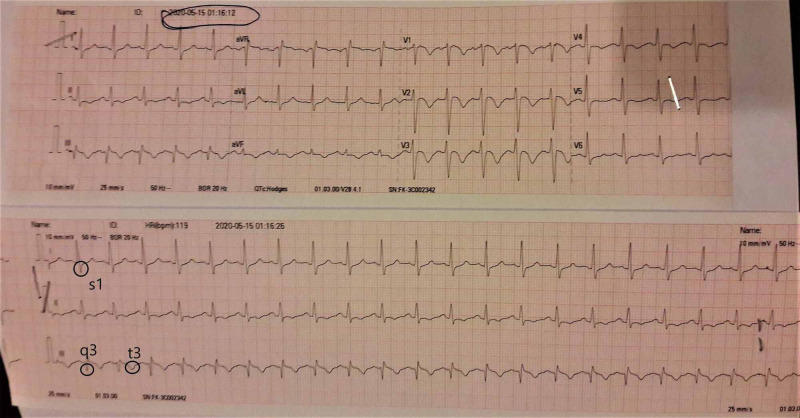
Electrocardiogram of a classical pattern S1Q3T3.

**Figure 5:**
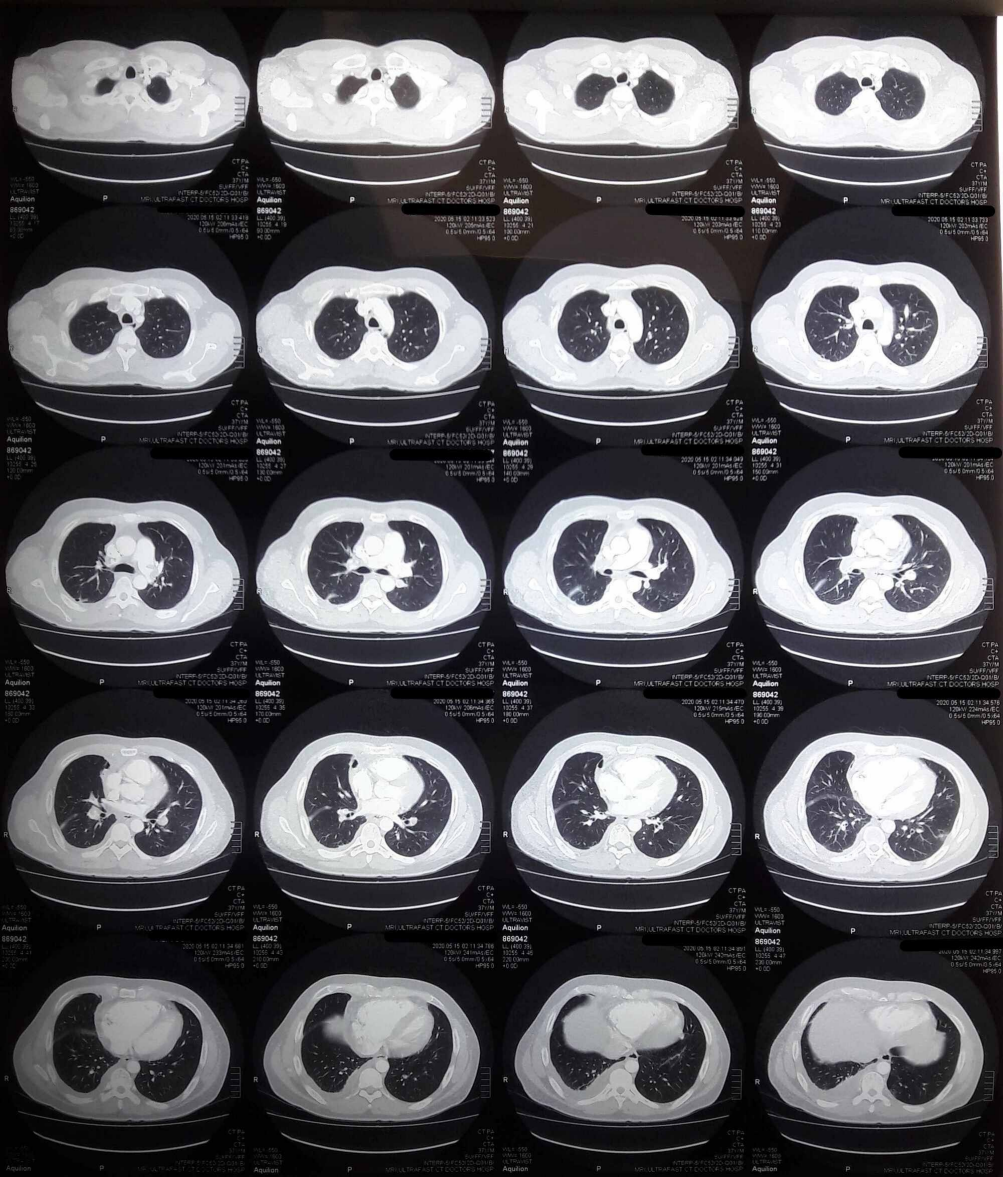
Chest high-resolution computed tomography (axial plane). Large right pleural effusion and infiltrates in the left lung.

Now, the patient presented with worsening dyspnea for the second time. Ultrasound Doppler scan of the right lower limb demonstrated deep vein thrombosis (DVT). Computerized tomography pulmonary angiogram (CTPA) showed bilateral occlusive pulmonary thromboembolism (Figure 1) involving the right pulmonary artery (Figure 2) and the left pulmonary artery (Figure 3). The electrocardiogram showed a classic appearance of the S1Q3T3 pattern in our case (Figure 4). HRCT performed showed persistent right-sided pleural effusion and compressive atelectasis in lower zones (Figure 5, Figure 6). The echocardiogram revealed a dilated right ventricle with systolic dysfunction and dilated pulmonary arteries. Anticoagulation with unfractionated heparin was started and the patient improved clinically in a few days. As part of the workup, investigations for blood thrombophilia were unremarkable. Deficiency of Anti-thrombin III, protein C, and protein S was ruled out and family history was not significant for any blood dyscrasias. Hence, common inherited hypercoagulable states were ruled out. Family history didn’t convey any thrombotic or metabolic risks. There was no history of prolonged immobility or trauma. No surgery was performed in the past three months or before. There was no history indicative of malignancy. Previous thromboembolism could not be ruled out due to a lack of required data. The patient had no hematological risk factors as well, for example, no history of heparin administration during the last presentation. There was no clinical evidence of renal, hepatic, and gastrointestinal pathologies that could lead to VTE. Hence, current TB infection and history of smoking stood out as the possible culprits. However, our patient was an ex-smoker. Hence, infection remained to be the sole apparent underlying risk factor for VTE.

 

## Discussion

TB infection manifests in many ways and leads to a range of symptoms according to the system it affects. Lung involvement, which is the predominant manifestation, contributes to pulmonary symptoms but is not strictly limited to cough with or without blood, chest pain, and breathing difficulty. In addition to the respiratory system, other systems can be affected including the gastrointestinal, bone, muscle, and central nervous system, known as extra-pulmonary TB. Hypercoagulability in patients with active TB has now started to be reported in the last few years. According to a study published in 2019, only 49 (0.6%) out of 7,905 patients with diagnosed TB were predisposed to the formation of thrombogenic profiles and complications such as deep venous thromboembolism (DVT), pulmonary embolism (PE), or both. A major proportion of the patients had PE as compared to the DVT, 42.9%, and 26.5%, respectively. The remaining number of patients (30.6%) had concurrent DVT and PE [5,6].

In 1950, the first-ever report was published which highlighted a strong correlation in patients with active TB and pulmonary embolism. A total number of 634 autopsies (24.3%) demonstrated PE in active TB compared to 23.1% incidence of PE in the entire series [7]. Individuals with active TB infection have almost as much risk of developing VTE as the ones with neoplasia [4]. Other pieces of literature have laid similar emphasis on the hemostatic changes in the background of TB infection resulting in hypercoagulability.

The patient in our case did not have any risk factors for thromboembolism apart from the newly diagnosed TB infection and former history of tobacco smoking. He was on treatment for TB for the last two weeks. Considering his age, background, absence of other active risk factors, occupation, and family history it was TB as the underlying cause of his PE.

## Conclusions

Based on the emerging literature, there is a potential association between TB infection and PE. This case report highlights the need to consider TB as a probable risk factor for thromboembolic events as much as other background pathologies like infection and neoplasia. Moreover, the risk of VTE should be taken into account during the management of patients with active TB, to prevent life-threatening events like PE.
